# Median Arcuate Ligament Syndrome Causing Nutcracker Syndrome and Left-Sided Varicocele

**DOI:** 10.7759/cureus.83177

**Published:** 2025-04-29

**Authors:** Sarth Shah, Niket Patel, Laurence Spitzer

**Affiliations:** 1 Radiology, Drexel University College of Medicine, Philadelphia, USA; 2 Radiology, University of Pennsylvania, Philadelphia, USA

**Keywords:** ct imaging, median arcuate ligament syndrome, nutcracker syndrome, sma compression syndromes, varicocele

## Abstract

Median arcuate ligament syndrome (MALS) is a rare vascular disorder caused by the compression of the celiac artery by the median arcuate ligament, leading to symptoms such as postprandial abdominal pain, weight loss, and nausea. While MALS typically affects the celiac artery, it can also result in downstream vascular complications, including dilatation of the superior mesenteric artery (SMA) to compensate for impaired blood flow, which can compress the left renal vein and cause nutcracker syndrome (NCS). This cascade may lead to secondary varicocele, characterized by dilatation of the pampiniform venous plexus.

We present a case of a 21-year-old male with abdominal pain, nausea, weight loss, and heaviness in his scrotum who was found to have a large left-sided varicocele on physical examination. Diagnostic imaging revealed MALS with celiac artery compression, SMA dilatation, and compression of the left renal vein, indicative of NCS. These findings suggest a unique vascular interplay, where SMA dilatation caused by collateral flow compensating for celiac artery obstruction contributed to secondary varicocele formation. The patient was informed of his diagnosis but ultimately declined surgical treatment.

This case underscores the importance of thorough diagnostic imaging and clinical evaluation in patients with rare overlapping vascular compression syndromes. It highlights the potential for a hemodynamic relationship between MALS, SMA dilatation, and NCS, contributing to the development of a varicocele. Further research is warranted to explore shared mechanisms and refine management strategies for such rare vascular presentations.

## Introduction

Vascular compression syndromes represent a group of conditions in which blood vessels are mechanically compressed by adjacent anatomical structures, leading to a spectrum of symptoms depending on the affected vessel. Among these syndromes, median arcuate ligament syndrome (MALS) and nutcracker syndrome (NCS) are particularly noteworthy due to their overlapping vascular dynamics and their potential to cause secondary complications such as varicocele. The coexistence of these conditions in a single patient remains exceedingly rare but provides valuable insights into the interplay of arterial and venous compressive mechanisms.

MALS involves the extrinsic compression of the celiac artery by the median arcuate ligament, resulting in ischemia and irritation of the celiac plexus. MALS affects 2 in 100,000 people, presenting with chronic postprandial abdominal pain, nausea, weight loss, and in some cases, vascular complications such as aneurysms or gastric ischemia [1]. The condition is diagnosed using a combination of clinical evaluation, duplex ultrasonography, and confirmatory imaging modalities such as computed tomography (CT) or magnetic resonance imaging (MRI) [2]. Treatment primarily involves surgical release of the ligament, which has been shown to improve symptoms and quality of life in most patients [3].

NCS, by contrast, is caused by compression of the left renal vein between the superior mesenteric artery (SMA) and the aorta, leading to venous hypertension [4]. The resulting pressure gradient often leads to hematuria, flank pain, and in males, varicocele - a dilation of the pampiniform plexus due to reflux into the gonadal vein [5]. Varicocele, while often idiopathic, is frequently secondary to venous compression syndromes like NCS, particularly when observed on the left side [6]. Diagnostic confirmation of NCS relies on imaging techniques such as Doppler ultrasonography or CT imaging, which assess and identify anatomical abnormalities [7].

MALS may play a substantial role in causing NCS. MALS involves a lack of perfusion through the celiac artery. This forces the SMA to compensate for perfusion to the organs of the foregut. This dilatation of the SMA causes NCS, which may cause the downfield effects of a varicocele [8]. The relationship between MALS and secondary conditions, such as NCS and varicocele, underscores the complexity of vascular compression syndromes. The resulting cascade highlights the need for a comprehensive diagnostic approach when patients present with overlapping symptoms of abdominal and scrotal vascular anomalies.

This report details the case of a 21-year-old male presenting with abdominal pain, significant weight loss, and a large left-sided varicocele. Imaging revealed compression of the celiac artery consistent with MALS, as well as SMA dilation and left renal vein compression indicative of NCS. This unique presentation emphasizes the necessity of a multidisciplinary approach for diagnosing and managing complex vascular compression syndromes. Furthermore, it illustrates the importance of identifying interconnected vascular anomalies to optimize patient outcomes and expand the understanding of these rare but significant conditions.

## Case presentation

We present a 21-year-old male who presented to the clinic with abdominal pain, nausea, weight loss, and a lump in his scrotum. The patient reported a history of abdominal pain for three to four years, mild difficulty gaining weight, nausea, postprandial discomfort, and a history of heaviness in his scrotum. A physical exam was performed, revealing hemodynamically stable vital signs, mild diffuse abdominal tenderness, and a large left-sided varicocele. Blood work was collected, demonstrating an unremarkable CBC, CMP, lipid panel, and inflammatory markers (Table 1). At this point, the differential included irritable bowel syndrome, microscopic colitis, inflammatory bowel disease, constipation, lactose intolerance, and infectious causes. After diet control for the removal of gluten and lactose failed to resolve symptoms, a stool test for bacterial antigens was ordered, which was negative for Escherichia coli, Helicobacter pylori, Clostridioides difficile, and Salmonella species. An esophagogastroduodenoscopy and colonoscopy were subsequently ordered, which revealed grade 1 hemorrhoids but were otherwise unremarkable. After diet control, stool testing for bacterial antigens, esophagogastroduodenoscopy, and colonoscopy revealed no findings or change in symptoms, a CT chest/abdomen with contrast was ordered.

**Table 1:** Lab work Pertinent values from CBC, fasting glucose, and CMP. LDL cholesterol is slightly elevated. Other values are within normal limits. TSH: thyroid-stimulating hormone; HDL: high-density lipoprotein; LDL: low-density lipoprotein; CBC: complete blood count; CMP: comprehensive metabolic panel

Parameters	Patient Values	Reference Range
White blood cell count	4.3	4.2-11.8 10^3^/uL
Hemoglobin	15.6	13.1-17.1 g/dL
Hematocrit	47.5	40.0-50.4 %
Platelet count	308	147-365 10^3^ uL
Glucose (fasting)	81	65-99 mg/dL
TSH	1.67	0.43-4.41 uIU/mL
Triglycerides	53	10-149 mg/dL
HDL cholesterol	42	>39 mg/dL
LDL cholesterol	109	<100 mg/dL
Sodium	141	136-145 mmol/L
Potassium	4.9	3.5-5.1 mmol/L
Chloride	104	98-107 mmol/L
Creatinine	0.8	0.7-1.3 mg/dL
Calcium	9.1	8.6-10.5 mg/dL
Alkaline phosphatase	61	20-130 U/L
Alanine transaminase	18	7-52 U/L
Aspartate transaminase	21	11-39 U/L

**Figure 1:**
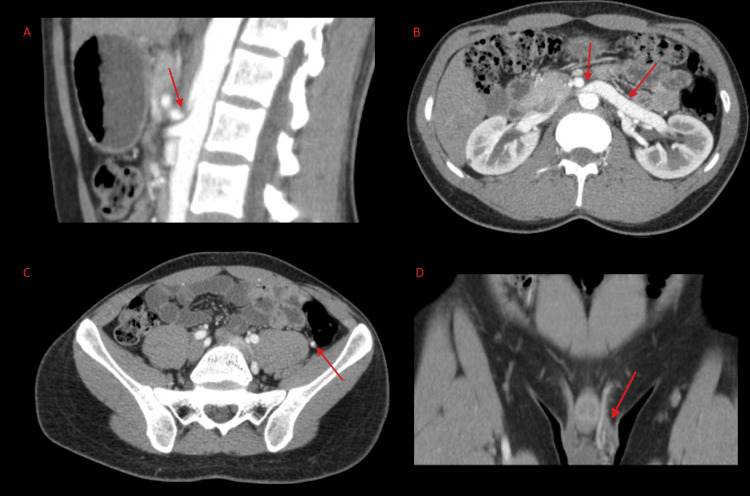
CT chest/abdomen with contrast (A) Sagittal CT abdomen with contrast demonstrating the median arcuate ligament compressing the celiac artery with apparent post-stenotic dilatation; arrow points to the stenosis point of the celiac artery. (B) Axial CT abdomen with contrast demonstrating a dilated SMA compressing left renal vein drainage, consistent with nutcracker syndrome; arrows point to the stenosis point of the left renal vein and dilatation of the left renal vein. (C) Axial CT abdomen with contrast demonstrating a dilated left spermatic vein compared to the right; arrow points to the dilated left spermatic vein. (D) Coronal CT abdomen with contrast demonstrating dilated pampiniform plexus veins, indicating a varicocele; arrow points to the dilated pampiniform plexus veins.

The CT scan reported rather unique and unusual findings: the celiac artery was experiencing compression by the median arcuate ligament, indicating MALS (Figure 1). The left pampiniform plexus veins were largely dilated, indicating a varicocele. The left spermatic vein was also hyperdense and enlarged, ascending to the left renal vein. The left renal vein was noticeably dilated compared to the right renal vein. The SMA was noticeably dilated and hyperdense, causing compression on the drainage of the left renal vein. This etiology suggests the SMA is likely enlarged due to the taxing of the collaterals between the SMA and the celiac artery because of the MALS compressing the celiac artery. This anatomy is consistent with the nutcracker phenomenon, which would cause the left-sided varicocele in our patient. The patient was informed of his likely diagnosis and referred to vascular surgery. The patient ultimately declined surgical treatment. The patient on follow-up is hemodynamically stable without life-threatening symptoms or signs.

## Discussion

The celiac artery branches into the left gastric, splenic, and common hepatic arteries. These vessels provide blood flow to the liver, stomach, spleen, pancreas, and upper duodenum. When the celiac artery is compressed, such as in MALS, these organs have reduced perfusion, causing pain and discomfort. However, the celiac artery shares various collaterals with the SMA that are hypothesized to provide continuous blood flow to the organs of the foregut to avoid ischemia [9]. If the celiac artery is compressed, the SMA will provide collateral blood flow via the inferior pancreaticoduodenal artery anastomosing with the gastroduodenal artery off the celiac artery [10]. This will cause the SMA to be dilated due to the need to provide increased blood. The expanded SMA can further cause compression of the left renal vein, which traverses beneath it. This is known as left renal vein entrapment, or NCS [7]. The entrapped left renal vein will cause blood to backflow through the spermatic vein and pampiniform plexus, causing a left-sided varicocele [4]. Varicoceles can cause a feeling of heaviness and a dull achy pain in the scrotum, as well as a visible swollen lump. It is often described as feeling like a “bag of worms,” and it is present in approximately 40% of males with infertility [11]. Varicoceles are recommended to be treated by varicocelectomy or embolization [12], but due to the underlying pathology of MALS and NCS, open surgery may be necessary [13]. Thus, it is important to recognize and diagnose MALS to guide treatment options.

MALS is a rare condition causing compression of the celiac artery and reduced blood flow to the abdominal organs, resulting in postprandial discomfort, nausea, abdominal pain, and weight loss. MALS is diagnosed through radiographic imaging such as MRI or CT with contrast [13]. After diagnosis, vascular surgery should be notified, and the recommended treatment option would be laparoscopic celiac artery decompression and release. A resolution of symptoms is seen in approximately 83% of patients, and recurrence of symptoms post-operation is as low as 18% [13]. After surgery, the taxing of blood from the SMA will be reduced, resolving NCS [8]. Resolution of NCS has been documented to resolve the varicocele in some cases without varicocele intervention and mitigate the chance of varicocele recurrence [14].

This clinical etiology is extremely rare, but it has been documented previously [8]. This diagnosis and pathophysiology were elucidated through CT imaging in previous studies, as in our case report. If the varicocele is treated via traditional approaches without treating the underlying MALS, the varicocele would recur as the elevated pressure on the spermatic vein persists, and it would not resolve the gastrointestinal symptoms our patient was experiencing. By using CT imaging, we were able to discover the cause of both the gastrointestinal symptoms and the heaviness in his scrotum. If the patient consented to the surgical release of MALS, this could have resolved all his symptoms [14]. This demonstrates the importance of recognizing rare etiologies of varicoceles through the use of radiographic CT/MRI imaging in order to guide treatment options and symptom management.

## Conclusions

MALS is a condition where the celiac artery is compressed by the median arcuate ligament. It results in decreased perfusion to abdominal organs, causing symptoms such as abdominal pain, postprandial discomfort, and weight loss. This rare condition is diagnosed through radiographic imaging, which can also reveal a myriad of other downstream effects caused by MALS, such as NCS with resultant varicocele. In this context, the development of a varicocele is not an isolated pathology but rather a secondary manifestation of altered vascular hemodynamics stemming from MALS-induced vascular compression. Recognizing this relationship emphasizes the need for comprehensive evaluation and imaging in patients presenting with varicoceles, especially when accompanied by abdominal symptoms. This unusual combination of vascular pathology has been rarely reported previously in the literature, making our case especially unique and of scientific value.

In our case study of a 21-year-old male, CT imaging was able to identify the cause of his abdominal pain, and unexpectedly, the cause of his varicocele. It was also able to elucidate how these seemingly unrelated symptoms are vascularly connected. This underscores the necessity of utilizing radiographic imaging and considering rare vascular pathologies to guide treatment options and therapeutic measures.
